# Comparative Safety, Effectiveness, and Outcomes of 10/12 Fr Versus 12/14 Fr Ureteral Access Sheaths and the Impact of Suction-Enabled Sheaths in Ureteroscopy

**DOI:** 10.7759/cureus.98734

**Published:** 2025-12-08

**Authors:** Mohamed Gad, Hesham H AbdelAziz

**Affiliations:** 1 Urology, Guy's and St Thomas' NHS Foundation Trust, London, GBR; 2 Urology, Al Soliman Hospital, Port Said, EGY

**Keywords:** flexible ureteroscopy, intrarenal pressure, postoperative sepsis, pre-stenting, suction uas, ureteral access sheath, ureteral injury

## Abstract

The choice of ureteral access sheath (UAS) size in flexible ureteroscopy (fURS) is pivotal in balancing endoscopic performance with ureteral safety. Larger sheaths (12/14 Fr) provide superior irrigation flow and intrarenal pressure control but raise concerns regarding ureteral trauma, particularly in unstented ureters. This PRISMA-aligned systematic review (January 2010-August 2025) synthesizes the available evidence comparing 10/12 Fr and 12/14 Fr UAS, both standard and suction-enabled, in terms of pressure modulation, irrigation efficiency, thermal regulation, ureteral injury, stricture formation, and infectious outcomes. Emerging data show that suction-assisted 12/14 Fr sheaths significantly lower rates of postoperative fever and sepsis without increasing ureteral injury when applied judiciously. Pre-stenting remains a key strategy for minimizing insertion-related trauma. The findings highlight an evolving clinical paradigm in UAS selection: shifting from a purely size-based choice to a physiology-driven approach that optimizes safety, visibility, and procedural efficiency.

## Introduction and background

Flexible ureteroscopy (fURS) for intrarenal stones has greatly benefited from the use of ureteral access sheaths (UAS). A UAS is a temporary coaxial conduit placed in the ureter to facilitate repeated instrument passage and improve irrigation outflow. By enhancing egress of irrigant, UAS use can help limit intrarenal pressure elevations during fURS, particularly under high-flow irrigation conditions [1]. In contemporary practice, 10/12 Fr and 12/14 Fr sheaths are the standard working sizes, with intermediate or larger diameters (e.g., 11/13 Fr or 14/16 Fr) selected selectively based on ureteral anatomy and surgeon preference. A larger-caliber sheath (12/14 Fr) offers superior irrigation and outflow, which can dramatically lower intrapelvic pressures compared to a smaller 10/12 Fr sheath [1]. However, the trade-off is a potentially higher risk of ureteral trauma with larger sheaths, especially in non-dilated ureters. Smaller sheaths (10/12 Fr or below) are gentler on the ureter but may not provide as much flow or pressure relief. This balance between access sheath size, irrigation efficiency, and ureteral safety has been a key consideration in fURS practice.

In recent years, a major innovation has been the advent of suction-enabled UAS (sUAS). Traditional sheaths are passive conduits, but newer sUAS actively aspirate fluid and stone debris during lithotripsy. By integrating continuous vacuum suction, these devices can evacuate laser-generated fragments and maintain low intrarenal pressures in real time. Early evidence suggests that suction-assisted systems are a “game changer” for fURS, improving the procedural trifecta: higher single-procedure stone-free rates, fewer complications, and reduced need for secondary interventions [2-4].

Despite these advancements, uncertainty persists regarding the optimal choice of UAS caliber and the true clinical impact of suction technology. Prior studies vary in design, outcome definitions, and methodologies, making direct comparison difficult. This systematic review therefore aims to synthesize current comparative evidence on how UAS size and suction capability influence intrarenal pressure, irrigation flow, ureteral injury, infectious complications, and overall procedural effectiveness during fURS.

Methods

This systematic review was conducted in accordance with the PRISMA 2020 reporting guidelines. A comprehensive literature search across PubMed, Embase, and Scopus was performed for studies published between January 2010 and August 2025, limited to English-language human research. Eligible studies were those that directly compared different UAS calibers, such as 10/12 Fr versus 12/14 Fr, or evaluated sUAS relative to standard non-suction sheaths during fURS. Both randomized and non-randomized comparative studies were included, while single-arm case series, cadaveric or animal experiments, and non-comparative narrative or scoping reviews were excluded. Two reviewers independently screened titles and abstracts before assessing full texts, with discrepancies resolved by consensus. In total, 18 studies encompassing over 3,200 patients met the inclusion criteria.

For each study, data were extracted on design, population characteristics, and key procedural outcomes, including irrigation flow dynamics, intrarenal pressure (IRP) measurements, UAS insertion success or failure, the severity of ureteral injuries (using standardized grading where reported), postoperative ureteral stricture rates, infectious outcomes (such as postoperative fever, urinary tract infection, or sepsis), and any clinical advantages attributable to sUAS. Owing to heterogeneity in methodology, outcome definitions, and IRP measurement techniques, a qualitative synthesis was performed rather than a pooled meta-analysis, although findings from existing meta-analytic work are referenced where appropriate. The primary objective of this review was to evaluate how UAS diameter and the presence or absence of suction influence safety and procedural efficacy in contemporary flexible ureteroscopy.

**Figure 1:**
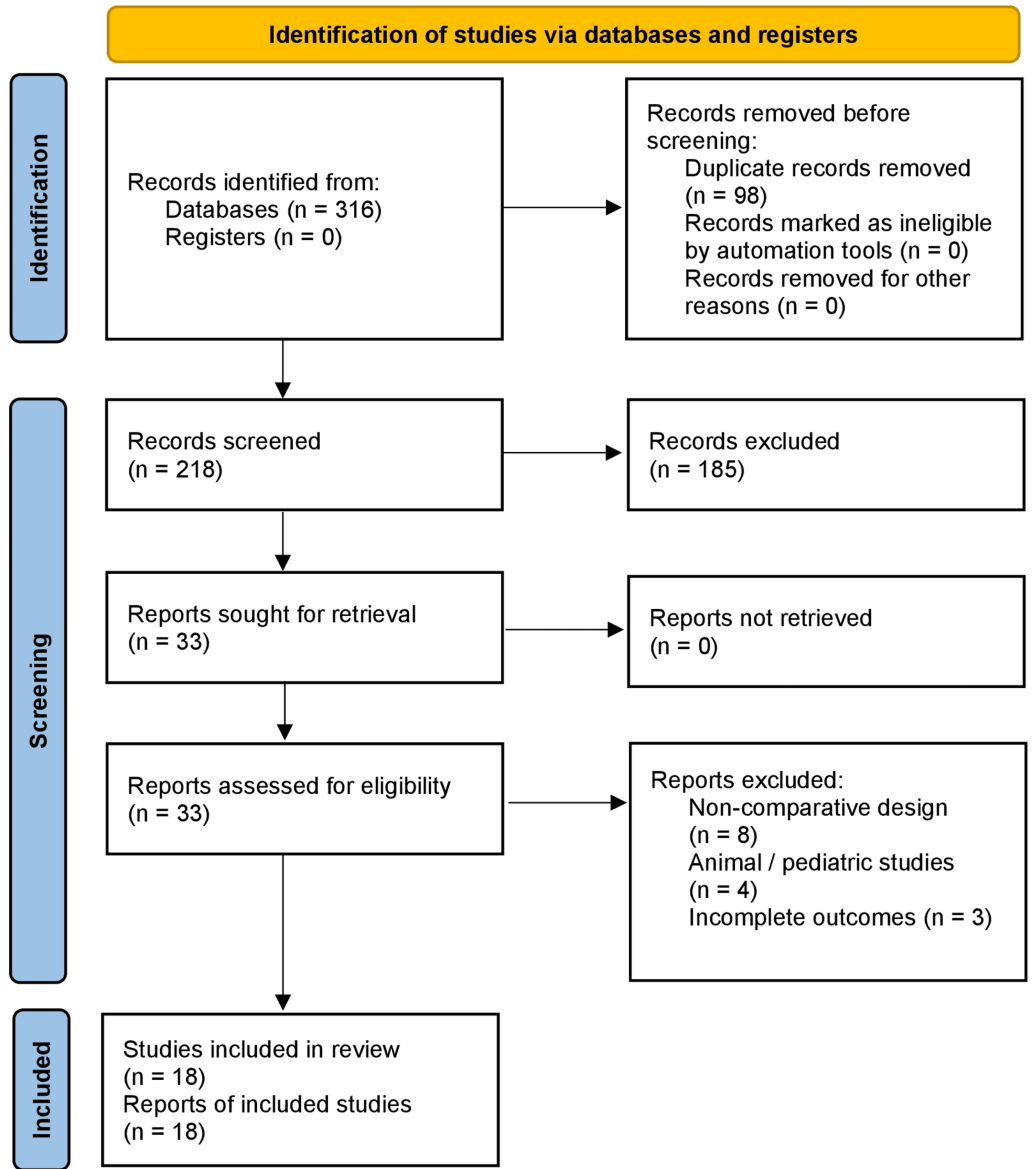
PRISMA 2020 flow diagram showing study identification, screening, eligibility assessment, and inclusion for this systematic review. A total of 316 records were identified through database searching. After removal of 98 duplicates, 218 records were screened, 33 full-text articles were assessed for eligibility, and 18 studies were included in the final qualitative synthesis.

The study selection process is summarized in Figure 1, presented in accordance with the PRISMA 2020 guidelines.

## Review

Intrarenal pressure and irrigation flow

Effect of UAS Caliber on Pressure and Flow

The caliber of the UAS exerts a direct and quantifiable influence on irrigation dynamics during fURS. In a controlled porcine kidney model, Wright et al. demonstrated that a 12/14 Fr sheath nearly doubled irrigation flow compared with a 10/12 Fr sheath (mean 33 mL/min vs 17 mL/min, p < 0.0001) [1]. This enhanced outflow translated into a substantial reduction in IRP, with the larger sheath maintaining mean pressures below ~40 cm H₂O even without instrumentation, whereas the smaller 10/12 Fr sheath yielded markedly higher values [1].

Under pressurized or high-flow conditions, such as during basketing or manual irrigation, the disparity becomes more pronounced. Intrapelvic pressure can surge to 118-121 cm H₂O with a 10/12 Fr sheath but remains near 29-31 cm H₂O with a 12/14 Fr sheath [1,2]. These experimental findings demonstrate that larger-caliber sheaths provide more efficient hydraulic decompression and better maintain pressures within physiologically safer ranges.

Clinical series corroborate these observations and consistently show that 12/14 Fr sheaths help sustain IRP below the generally accepted safety threshold of ~40 cm H₂O (~30 mmHg) [3,4]. Additional prospective and real-world studies support these physiologic advantages, reporting sustained low pressures and improved visualization with larger or suction-enabled sheaths in contemporary practice [5]. Conversely, smaller sheaths, or the absence of one, permit dangerous rises in IRP during high-flow phases, predisposing to forniceal backflow, mucosal ischemia, and postoperative infective sequelae. Recent pressure-monitoring studies using digital pressure-sensing ureteroscopes have further validated these flow-dependent pressure differences in vivo [6].

Impact of Suctioning UAS

The evolution from passive to sUAS represents a major advancement in intrarenal pressure management. By actively aspirating irrigation fluid and particulate debris, sUAS establish a continuous low-pressure environment that counterbalances inflow. Real-time pressure monitoring with a sensor-equipped flexible ureteroscope demonstrated that sUAS maintain median renal pelvic pressures around 22 mmHg (~30 cm H₂O) even under aggressive irrigation (250 mmHg pressure bag) [7].

Clinical evidence extends this observation, showing that sUAS can maintain IRPs below 40 mmHg during most of the operative time, including in cases involving large stone burdens [7,8]. Surgeons further report qualitative benefits, such as improved endoscopic visibility, faster clearance of stone dust, and preserved deflection, owing to continuous removal of turbid fluid [2]. From a physiological standpoint, this steady-state decompression mitigates pyelovenous and pyelolymphatic backflow, thereby reducing the risk of postoperative infection and urosepsis. Similar findings have been demonstrated in multi-institutional experience and prospective surveillance evaluating suction systems in routine clinical practice [8,9].

Multicenter randomized and cohort data have reinforced these pressure-lowering and visualization advantages with flexible sUAS [10-12]. Accordingly, a 12/14 Fr sheath equipped with active suction appears to provide the optimal balance between access, visibility, and safety across a range of procedural conditions.

Temperature Considerations

Beyond pressure modulation, irrigation through the UAS functions as a thermal buffer during laser lithotripsy. Elevated temperatures exceeding ~43°C pose a risk of urothelial injury and coagulative necrosis. Experimental work by Multescu et al. [6] showed that with continuous irrigation at ~40 mL/min via a 10/12 Fr sheath, calyceal temperatures remained between 36°C and 42°C during high-power holmium:YAG activation (up to 16 W) [13].

These findings demonstrate that adequate irrigation and outflow are generally sufficient to dissipate laser-generated heat under standard operative conditions. Modern high-power holmium and thulium fiber laser systems (20-30 W) have similarly shown no evidence of thermal injury when used with standard or sUAS [13]. Suction systems may offer additional benefit by facilitating continuous exchange of warm effluent with cooler irrigant, thereby stabilizing intrarenal temperature gradients throughout lithotripsy [14].

Ureteral trauma, sheath insertion, and stricture

Ureteral Wall Injury Rates

The main drawback of UAS use is the potential for iatrogenic ureteral injury. The ureter must accommodate the outer diameter of the sheath, creating a risk of mucosal tears or ischemia from radial stretch or friction. Multiple studies have evaluated these injuries using standardized endoscopic grading systems. In a landmark prospective study of 359 patients, Traxer and Thomas [7] directly inspected the ureter after fURS with a 12/14 Fr sheath and found ureteral wall injury in 46.5% of cases [15]. Although most were low grade, 13.3% involved deeper injury into the smooth muscle layer [15]. Because smaller sheaths were not used in that cohort, the degree to which injury rates scale with sheath size could not be assessed in that study.

Subsequent randomized evidence confirms that downsizing the sheath reduces trauma. In a trial of 320 patients, Aykanat et al. [8] reported ureteral injury of any grade in ~39% of cases with a 12/14 Fr sheath versus ~24% with a 9.5/11.5 Fr sheath (p = 0.013) [16]. High-grade injuries were also more common with the larger sheath (11.9% vs 5.0%) [16]. Nearly all injuries in both groups were successfully managed with temporary stenting, and long-term sequelae were rare. Although ureteroscope diameter and technique also contribute to injury risk, sheath size remains one of the most influential procedural variables.

Role of Pre-stenting

Preoperative ureteral stenting, typically for one to two weeks, is well established as a protective factor during UAS placement. In one study, the absence of a pre-existing stent increased the risk of high-grade injury sevenfold [15]. Nearly all severe injuries occurred in unstented ureters.

A 2022 meta-analysis by Law et al. similarly found that pre-stented patients had significantly higher insertion success rates and a 31% lower risk of ureteral injury compared with non-stented patients (RR 0.69; 95% CI 0.50-0.96) [9]. These findings underscore the importance of pre-stenting, particularly if a larger (12/14 Fr) sheath is planned or if the ureter is suspected to be narrow. In practical terms, pre-stenting can convert an “up-sizing” from 10/12 to 12/14 Fr from a risky endeavor into a routine one by achieving sufficient passive dilation. Thus, whenever feasible, placing a stent prior to fURS can significantly mitigate insertion force and ureteral trauma.

Insertion Failure Rates

Even with careful technique, there will be cases where a UAS cannot be advanced into the ureter, particularly in unstented or narrowly anatomized ureters. Larger sheaths are predictably more prone to insertion failure. Reported failure rates for 12/14 Fr UAS in non-pre-stented (“virgin”) ureters range from approximately 13% to 20% [10]. In contrast, 10/12 Fr sheaths fail to pass in only about 5%-10% of cases (8.8% in one series) [10]. In practical terms, the smaller sheath succeeds the vast majority of the time, whereas a 12/14 Fr sheath may fail in up to one in five attempts when the ureter has not been dilated.

When the UAS cannot be advanced safely, the surgeon must either proceed without a sheath or abandon the attempt and stage the procedure after ureteral stenting. Pre-stenting again plays a central role; it significantly improves first-pass UAS insertion success (RR ~1.09, p < 0.0001) [9]. Some prospective comparative data also indicate that sheath design and flexibility influence insertion characteristics, with certain flexible sUAS demonstrating favorable insertion profiles in selected series [17].

Overall, a 10/12 Fr sheath is more likely to traverse a tight or naïve ureter, whereas a 12/14 Fr sheath offers better outflow and intrarenal pressure control when it can be placed. Clinically, the decision represents a balance between achieving lower pressure and better visibility (favoring larger sheaths) and maximizing the likelihood of safe, atraumatic insertion (favoring smaller sheaths or routine pre-stenting).

Ureteral Stricture Formation

A critical long-term outcome is whether UAS-related injuries translate into ureteral strictures. Fortunately, clinically significant strictures remain uncommon with contemporary UAS use. In the randomized trial referenced above, one-year imaging revealed an overall stricture rate of 1.6% (5 of 320 patients) [16]. Four strictures (2.5%) occurred in the 12/14 Fr group versus one case (0.6%) in the smaller-sheath group, but this difference was not statistically significant [16]. The single stricture in the smaller group occurred in a patient who sustained an insertion-related injury despite downsizing.

Other large series corroborate these findings. A study reported no long-term strictures attributable to even high-grade injuries, with all cases healing fully after short-term stenting [15]. Multicenter data likewise report post-fURS stricture rates consistently below 2%, with most strictures linked to unrecognized perforation or severe injury during access.

Thus, while higher UAS size and lack of pre-stenting increase acute injury risk, they have not led to a corresponding rise in strictures. Most UAS-related mucosal injuries are self-limited and heal without fibrosis if appropriately managed (e.g., short-term stenting). Nonetheless, the principle of atraumatic technique remains paramount: minimizing force during sheath insertion and respecting ureteral size limits will further reduce the already low risk of stricture formation.

Infectious outcomes and the impact of suction

One of the most clinically significant advantages of larger and especially sUAS is the reduction in infectious complications during fURS. Elevated intrarenal pressure has been strongly implicated in postoperative fever and sepsis due to pyelovenous and pyelolymphatic backflow of bacteria, endotoxins, and debris. By lowering intrapelvic pressure, UAS, particularly sUAS, have been associated with fewer postoperative infectious events.

Several comparative studies support this trend. Zhu et al. demonstrated a postoperative fever rate of 13.9% with standard UAS compared to 5.5% with suction UAS (p = 0.009), with urosepsis occurring in 6.7% vs 1.8%, respectively (p = 0.03) [2]. A meta-analysis by Kayano et al. (seven studies, n = 1,746) similarly showed that suction sheaths reduced overall complications by almost half (risk ratio ≈ 0.49) and decreased febrile UTI rates by approximately 63% (RR 0.37) [3]. Although severe events such as septic shock remain rare in all groups, the consistent reduction in febrile morbidity and SIRS across studies is clinically meaningful. Real-world observational data further reinforce these findings, particularly in high-risk or high-pressure procedural settings [4,11].

Suction systems also appear to confer secondary clinical benefits. In a comparative cohort study, Wang et al. reported a shorter mean hospital stay for patients treated with suction UAS (1.6 days) compared with standard UAS (~2.3 days), a difference attributed largely to fewer postoperative fevers and faster recovery [11]. Although not universally statistically significant, this favorable trend has been observed across multiple patient subsets, especially among those with infected stones or larger stone burden.

Reductions in unplanned healthcare utilization mirror these outcomes. One study reported lower 30-day readmission rates in the suction group, reflecting fewer returns for febrile illness or presumed urosepsis. A prospective multicenter experience involving nearly 400 suction-assisted cases reported no episodes of postoperative sepsis requiring escalation of care [4], emphasizing the robustness of low-pressure environments during fURS.

Importantly, suction-enabled systems achieve these advantages without increasing ureteral trauma. Comparative studies, including those by Kayano et al., found no significant difference in ureteral injury rates between suction and standard UAS [3]. Some authors have suggested that continuous evacuation of irrigant and stone dust may additionally reduce postoperative mucosal edema and discomfort. In the series by Wang et al., ureteral injury rates were comparable between suction and standard sheaths (~17% vs 19%, all low-grade), with no ureteral perforations in either group [11].

An added advantage repeatedly noted in the literature is improved early stone clearance. Active fragment aspiration enhances removal of fine particulate “dust,” resulting in 10%-15% higher postoperative day 1 stone-free rates in several studies, although longer-term outcomes converge once secondary procedures are accounted for [3,4]. This early efficiency may reduce the need for auxiliary interventions in selected patients.

Taken together, current evidence strongly supports sUAS as a valuable adjunct in contemporary fURS. Their ability to maintain low intrarenal pressure, reduce febrile complications, and improve early stone-free rates, without increasing ureteral injury risk, makes them particularly advantageous in high-flow, high-burden, or infected-stone scenarios. Appropriate sheath selection, combined with selective pre-stenting, remains essential to optimizing both safety and procedural performance.

Discussion

fURS has evolved significantly with the wider adoption of UAS, and contemporary evidence consistently demonstrates the physiologic advantages of optimizing outflow to control intrarenal pressure, maintain temperature stability, and reduce postoperative infectious morbidity. Larger-caliber sheaths and suction-enabled devices both improve fluid dynamics, enhancing visualization and reducing the risk of pressure-related complications during lithotripsy [1-14].

These trends are consistent across randomized trials and large multicenter cohorts evaluating current UAS platforms. Recent RCTs and prospective studies assessing flexible and navigable suction-enabled sheaths have demonstrated significantly lower intrarenal pressure profiles, more efficient clearance of irrigant and stone debris, and improved endoscopic visibility compared with conventional UAS systems. Importantly, these physiologic advantages appear to translate into meaningful clinical benefits, including reduced postoperative fever and urosepsis rates, higher single-session stone-free rates, and fewer unplanned readmissions across both high- and low-stone-burden cases. Multi-institutional real-world experience further reinforces that suction systems can achieve these benefits without increasing ureteral trauma when appropriately sized and inserted, particularly in pre-stented or non-dilated ureters. Collectively, this growing body of mechanistic and clinical evidence underscores a shift toward physiology-driven ureteroscopic practice, where maintaining low-pressure environments through optimized sheath design is central to procedural safety and efficiency [12-18].

Only a qualitative synthesis was performed, as heterogeneity in study design, patient selection, outcome definitions, and intrarenal pressure measurement techniques precluded a reliable pooled meta-analysis. Most comparative data remain observational, with limited randomized evidence specifically evaluating sheath diameter or suction technology. Although study quality and bias were considered conceptually, no formal appraisal tool (ROB-2 or ROBINS-I) was applied, given the narrative methodology of this review. Restriction to English-language publications may introduce language and publication bias.

Additional limitations arise from variability in follow-up duration, particularly for long-term outcomes such as stricture formation, and from rapid technological evolution, as newer pressure-monitoring ureteroscopes and flexible suction-enabled platforms may outperform earlier devices. Operator experience, irrigation pressures, stone characteristics, and pre-stenting practices also influence outcomes and contribute to heterogeneity across studies.

Despite these constraints, the consistency of physiologic and clinical trends across diverse study designs strengthens the conclusion that adequate outflow, whether achieved through larger-caliber sheaths or suction-enabled platforms, is central to safe and effective retrograde intrarenal surgery. Future research should prioritize standardized IRP reporting, long-term stricture surveillance, and multicenter randomized comparisons of sUAS versus traditional UAS to refine optimal sheath selection and patient pathways.

## Conclusions

UAS are now an indispensable adjunct in modern fURS, yet their optimal use requires careful balancing of irrigation efficiency and ureteral safety. Larger sheaths (12/14 Fr) offer clear advantages in outflow and intrarenal pressure control, but they remain technically more demanding to insert and carry a higher risk of ureteral wall injury in non-dilated or narrow ureters. Careful patient selection and preparation, particularly preoperative stenting in cases where a larger sheath is anticipated, can mitigate these challenges and support safe up-sizing when appropriate.

sUAS have further advanced intrarenal pressure control by providing continuous low-pressure irrigation and efficient fragment evacuation. Importantly, their benefits extend across sheath calibers: both 10/12 Fr and 12/14 Fr sheaths demonstrate improved visualization and reduced infectious morbidity when paired with suction compared to their non-suction counterparts. This distinction is clinically relevant, as many surgeons rely primarily on 10/12 Fr sheaths due to their easier insertion profile, especially in non-pre-stented ureters. In these scenarios, the option of 10/12 Fr with suction provides a practical balance between safety and pressure management. An individualized approach remains essential. A 12/14 Fr suction sheath in a pre-stented ureter offers maximal efficiency, while smaller sheaths, or staged dilation, are preferable in tighter or naïve ureters. As pressure-sensing scopes and next-generation suction systems continue to evolve, thoughtful sheath selection will remain central to achieving optimal visualization, efficient fragmentation, and minimal patient morbidity. Collectively, current evidence supports that judicious use of both standard and sUAS enhances the safety and effectiveness of retrograde intrarenal surgery.
